# Perceptual training modifies temporal sensitivity and a sense of agency

**DOI:** 10.3389/fpsyg.2023.1136365

**Published:** 2023-05-05

**Authors:** Agnese Venskus, Peter L. T. Gooding, Gethin Hughes

**Affiliations:** ^1^School of Human Sciences, University of Greenwich, London, United Kingdom; ^2^Department of Psychology, University of Essex, Colchester, United Kingdom

**Keywords:** perceptual training, temporal sensitivity, temporal binding window, sense of agency, neuroplasticity

## Abstract

Perceptual training has been argued to be a potential means to modify temporal sensitivity (the ability to detect a time-based discrepancy between two stimuli) with previous studies providing preliminary evidence that perceptual training can lead to increased temporal sensitivity. However, previous studies have not employed a control group and therefore cannot rule out the possibility that the observed effects are due to repeated completion of the task, rather than the training itself. Moreover, despite temporal sensitivity being suggested to be an important aspect of the sense of agency, the effects of perceptual training on the sense of agency have not been explored. Therefore, this study aimed to explore the effects of perceptual training on the sense of agency and replicate previously observed effects on temporal sensitivity while utilizing a more rigorous methodology. Given the existing literature, it was predicted that the sense of agency and temporal sensitivity will be enhanced following perceptual training. Temporal sensitivity was only weakly modified by perceptual training when compared to the control condition. Sense of agency was significantly modulated by perceptual training, over and above the control condition. This study's findings present novel evidence indicating that perceptual training can influence high-level processes such as the sense of agency and temporal sensitivity.

## 1. Introduction

Temporal grouping of sensory information is subjected to an individual's temporal sensitivity, that is, the ability to recognize a discrepancy in time between the instances of two stimuli (Colonius and Diederich, 2004). Existing research (Stevenson et al., 2014; Cecere et al., 2015; Ha et al., 2017; Ferri et al., 2018) shows that audio–visual temporal sensitivity varies significantly between individuals. Recent literature reviews (Hirst et al., 2020; Keil, 2020) have demonstrated that temporal sensitivity can be examined by measuring the temporal binding window (TBW). The TBW is defined as the window within which the integration of incoming multisensory information takes place. The most common way to measure the TBW is through a double-flash illusion task (Hirst et al., 2020; Keil, 2020). In this task, an individual is presented with the simultaneous occurrence of a visual (flash) stimulus and an auditory (beep) stimulus. Following a variable delay, a second auditory (beep) stimulus is presented. When both beeps are within the individual's TBW, the flash is integrated with both beeps. This then results in the perception of an illusionary second flash. As such, the delay at which an individual no longer perceives two flashes is taken as the width of their TBW and acts as an index of their temporal sensitivity. Temporal sensitivity has been suggested to be involved in various higher cognitive processes such as the sense of agency (SoA; Venskus et al., 2021).

The sense of agency (SoA) refers to the feeling of being in control of one's actions and their associated outcomes (Moore, 2016) and appears to be influenced by temporal sensitivity (Shanks et al., 1989; Knoblich and Kircher, 2004; Sato and Yasuda, 2005; Farrer et al., 2008, 2013; Kawabe et al., 2013; Venskus et al., 2021). Experience of SoA is reduced when there is an increase in the interval between an action and a resulting sensory outcome (Shanks et al., 1989). This relationship seems to depend on the temporal grouping of actions and outcomes (Kawabe et al., 2013). Moreover, Farrer et al. (2013) hypothesized that an individual experiences a greater SoA when an action and integrated outcome occur within a specific temporal interval. Their study required participants to press a button (action) that caused the appearance of a circle on a screen (outcome) over numerous occasions with various time delays. Participants were then required to report their SoA over the outcome. The results indicated that individuals felt greater SoA for the outcome where the delay was shorter and vice versa. Consequently, this supports the notion that SoA depends on the temporal relationship between action and outcome and, therefore, temporal sensitivity. Direct support for the relationship between SoA and temporal sensitivity is provided by Venskus et al. (2021). In this study, researchers used various tasks (simultaneity judgment task and double-flash illusion) to assess temporal sensitivity and a judgment of agency task, adapted from Farrer et al. (2013) study, to assess SoA. Results indicated that a wider TBW was associated with a wider SoA window.

Recently, temporal sensitivity has been shown to be modifiable via perceptual training by shifting the point of subjective simultaneity (PSS), the discrepancy at which two stimuli are perceived as simultaneous, toward the optimum (Powers et al., 2009; Stevenson et al., 2013; De Niear et al., 2018; Zerr et al., 2019). More precisely, the width of the TBW was shown to be reduced following perceptual training. Perceptual training involves judging stimuli as simultaneous or non-simultaneous with performance-based feedback being provided. However, previous studies have not employed a control group. As such, it remains possible that the observed reduction in TBW following perceptual training may not be due to the perceptual training itself but instead due to the repeated completion of the task. Therefore, the current study introduced a control condition. More specifically, in the experimental sample, participants' TBW was assessed before and after two sessions of perceptual training, while in the control group, the perceptual training days were replaced by rest days.

Moreover, if temporal sensitivity is related to SoA, one could hypothesize that enhancing temporal sensitivity via perceptual training could potentially modify SoA. This would clarify whether perceptual training can affect higher cognitive processes (i.e., SoA) in a similar way as it affects lower cognitive processes (i.e., temporal sensitivity). This study aimed to provide initial evidence to address this possibility.

## 2. Method

### 2.1. Participants

#### 2.1.1. Experimental sample

The experimental sample consisted of 75 student volunteers from the University of Essex, who were recruited via the University research advertisement websites (i.e., SONA) with course credits as reimbursement. All participants had normal or corrected to normal vision and hearing to avoid these variables influencing the tasks. Participants gave their informed consent before taking part in the study. The study was approved by the local ethics committee and was conducted in accordance with the ethical standards of the 1964 Declaration of Helsinki and was approved by the University of Essex‘s Faculty Ethics Subcommittee (departmental reference no: ETH2021-0206). Data were made accessible on a public repository, OSF, *via* the following link: https://osf.io/u4x8v/?view_only=7802a3d16c8d4ad19044996769b704fa.

#### 2.1.2. Control sample

The control sample consisted of 20 volunteers recruited *via* social media platforms with £20 as reimbursement. All participants had normal or corrected to normal vision and hearing to avoid these variables influencing the tasks. Participants gave their informed consent before taking part in the study. The study was approved by the local ethics committee and was conducted in accordance with the ethics standards of the 1964 Declaration of Helsinki and was approved by the University of Essex‘s Faculty Ethics Subcommittee (departmental reference no: ETH2021-0206). Data were made accessible on a public repository, OSF, *via* the following link: https://osf.io/u4x8v/?view_only=7802a3d16c8d4ad19044996769b704fa.

### 2.2. Data exclusion

#### 2.2.1. Experimental sample

A total of 55 datasets were removed from the double-flash illusion and 26 datasets were removed from the judgment of agency task. In the double-flash illusion, 35 datasets were excluded after being completed online using Mac devices, which distorted the task, such that only one tone was presented. This meant that participants could not experience the illusion. Furthermore, 20 datasets were excluded due to poor fit of the psychometric sigmoid function (R^2^ < 0.6); the R^2^ value shows how well the data fit the model, with a higher R^2^ value representing smaller differences between the observed data and the fitted data and hence a better fit (Cecere et al., 2015) and/or incomplete data. This left behind 20 datasets that were included in the analysis for temporal sensitivity. In the judgment of the agency task, all 26 excluded datasets were removed from further analysis due to poor fit of the psychometric sigmoid function (R^2^ < 0.6) or/and incomplete data. This left behind 49 datasets that were included in the analysis for the sense of agency.

A sensitivity power analysis was conducted using G^*^power (Faul et al., 2007). This approach used here was retrospective as the sample size was limited to a certain number due to the time available for the data collection. For temporal sensitivity, G^*^power was populated with a conventional α-level of 0.05, the available sample size of 20, and a conventional power of 80%. A sensitivity power analysis showed that our sample size had 80% power to detect large effect sizes for *t*-tests (dz = 0.66, α = 0.05, two-tailed). For SoA, G^*^power was populated with a conventional α-level of 0.05, the available sample size of 49, and a conventional power of 80%. A sensitivity power analysis showed that our sample size had 80% power to detect medium effect sizes for *t*-tests (dz = 0.41, α = 0.05, two-tailed).

#### 2.2.2. Control sample

From the control sample, 10 datasets were removed from the double-flash illusion, and five datasets were removed from the judgment of agency task. In the double-flash illusion, six datasets were excluded after being completed online using Mac devices, which distorted the task, and four datasets were excluded due to poor fit of the psychometric sigmoid function (R^2^ < 0.6) or/and incomplete data. This left behind 10 datasets that were included in the analysis for temporal sensitivity. In the judgment of the agency task, all five excluded datasets were removed from further analysis due to poor fit of the psychometric sigmoid function (R^2^ < 0.6) or/and incomplete data. This left behind 15 datasets that were included in the analysis for the sense of agency.

A sensitivity power analysis was conducted using G^*^power (Faul et al., 2007). This approach used here was retrospective as the sample size was limited to a certain number due to the time available for the data collection. For temporal sensitivity, G^*^power was populated with a conventional α-level of 0.05, the available sample size of 10, and a conventional power of 80%. A sensitivity power analysis showed that our sample size had 80% power to detect very large effect sizes for *t*-tests (dz = 1.00, α = 0.05, two-tailed). For SoA, G^*^power was populated with a conventional α-level of 0.05, the available sample size was 15, and a conventional power of 80%. A sensitivity power analysis showed that our sample size had 80% power to detect large effect sizes for *t*-tests (dz = 0.81, α = 0.05, two-tailed).

### 2.3. Design

#### 2.3.1. Experimental sample

For the experimental condition, the independent variable in the study was perceptual training with two conditions (pre-perceptual training and post-perceptual training). The dependent variables were the width of the TBW and the width of the SoA window.

#### 2.3.2. Control sample

For the control condition, the independent variable in the study was the number of times the tasks were performed with two conditions (initial and repeat). The dependent variables were the width of the TBW and the width of the SoA window.

### 2.4. Apparatus/materials

#### 2.4.1. Double-flash illusion (a measure of TBW)

**Figure 1:**
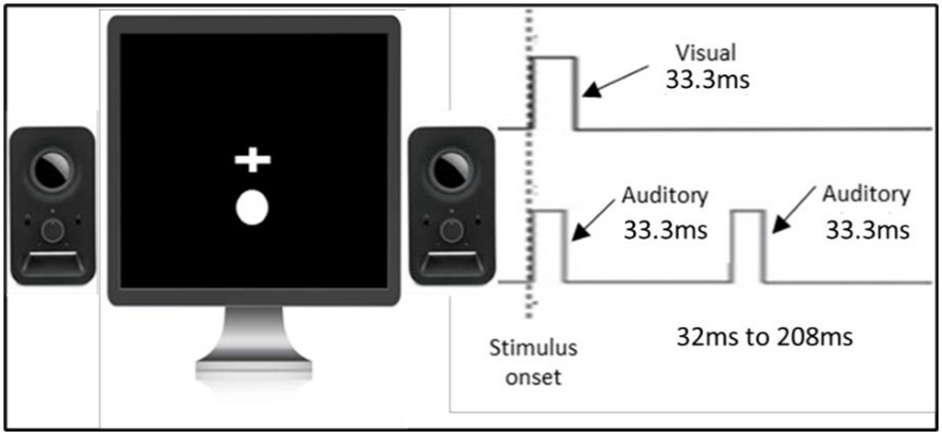
Paradigm of the double-flash illusion.

The double-flash illusion used was the same as that in the study of Cecere et al. (2015) (see Figure 1). The task was programmed and controlled by the INQUISIT Millisecond software package 5.0 (Millisecond Software, 2016) on an LCD monitor with a refresh rate of no <60 Hz. Visual and auditory stimuli were 33.3 ms in duration to ensure compatibility with the majority of monitors (60Hz refresh rate, 16.6 ms). Visual stimuli were in the form of a white circle with a diameter of 2.5 cm located 1 cm below the fixation cross that was positioned in the center of the screen. This stimulus setup was chosen as it has been shown that tasks involving multisensory integration are optimized when visual stimuli are displayed in peripheral vision (Shams et al., 2002). Auditory stimuli were in a form of a 3500 Hz pure tone. Each trial started with a white fixation cross in the center of the monitor that remained on the screen throughout the trial. On each trial, visual and auditory stimuli were presented simultaneously, and after a variable stimulus onset asynchrony (randomly chosen between 32 ms and 208 ms, in steps of 16 ms), a second auditory stimulus was presented. These particular stimulus onset asynchronies were also chosen to synchronize the stimulus timing with the refresh rate of the screen (60 Hz, 16.6 ms). Participants performed one block. Each stimulus onset asynchrony was presented 26 times, totalling to 312 trials. Participants were instructed to fixate on the fixation cross and report whether they perceived one or two flashes by pressing the keys ‘1‘ or ‘2‘, respectively.

#### 2.4.2. Judgment of agency (a measure of SoA)

**Figure 2:**
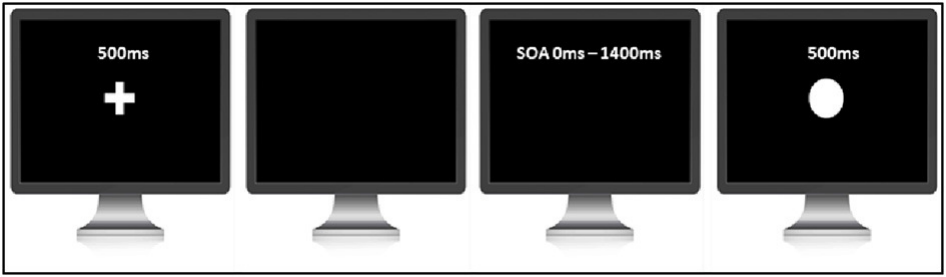
Paradigm of the judgment of agency task.

The judgment of agency tasks was adapted from Farrer et al. (2013) (see Figure 2). The task was programmed and controlled by the INQUISIT Millisecond software package 5.0 (Millisecond Software, 2016). Each trial started with a white fixation cross in the center of the monitor. After a delay of 500 ms, the fixation cross disappeared, signaling the beginning of the trial. After the cross disappeared, participants were asked to press the space bar on the computer keyboard whenever they wanted. Once participants pressed the key, a circle of 2.5 cm in diameter was displayed in the center of the screen for 500 ms with 11 possible delays ranging from 0 to 1400 ms in steps of 140 ms. The task consisted of two blocks with each delay being presented 10 times in random order. In total, participants completed 220 trials. Participants were required to judge if the appearance of the circle was caused by their button press, or if the computer had triggered the circle to appear. Participants were told that on some trials, the computer would cancel their button press and re-trigger the appearance of the circle at a random interval. Participants needed to press the key ‘1‘ if they thought that it was most likely they triggered the circle to appear and key ‘2‘ if they thought that it was most likely computer triggered the circle to appear. This response approach was chosen over that of Farrer et al. (2013), where participants were given three choices (i.e., full control, partial control, and no control), to avoid participants opting for the partial control if not fully confirmed. Such partial control responses would complicate the calculation of the time window of SoA via the fitting of a sigmoid function.

#### 2.4.3. Perceptual training

**Figure 3:**
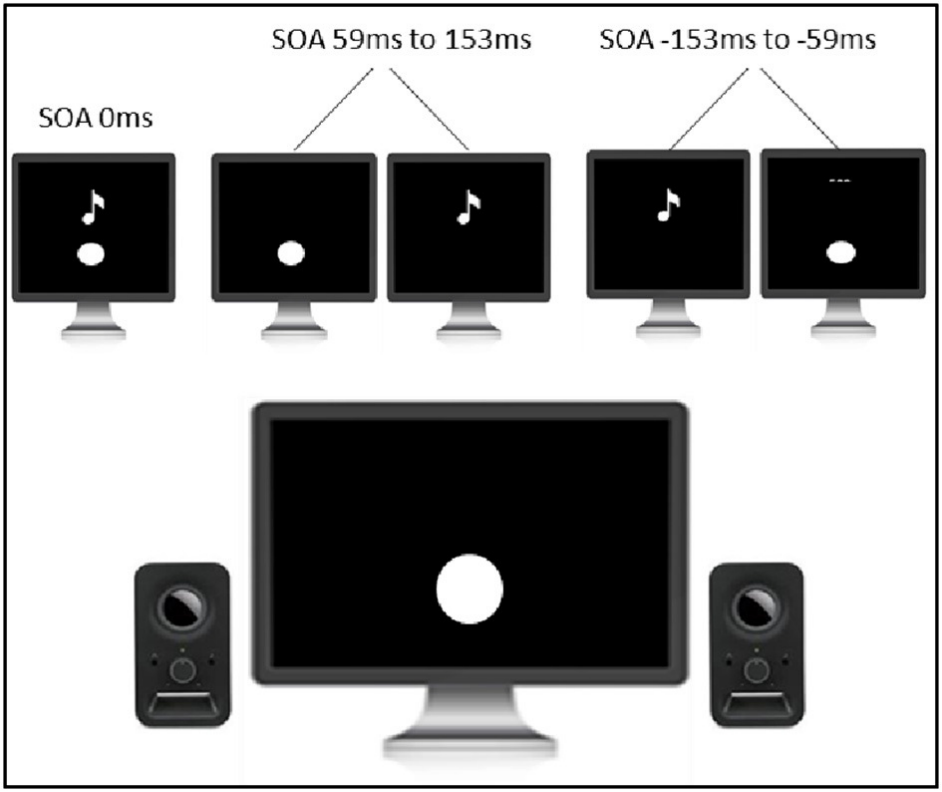
Paradigm of the perceptual training.

Perceptual training consisted of a simultaneity judgment task with feedback (see Figure 3). The task was programmed and controlled by the INQUISIT Millisecond software package 5.0 (Millisecond Software, 2016) on an LCD monitor with a refresh rate of no <60 Hz. Visual and auditory stimuli were 33.3 ms in duration to ensure compatibility with the majority of monitors (60 Hz refresh rate, 16.6 ms). Visual stimuli were in the form of a white circle with a diameter of 2.5 cm located 1 cm below the fixation cross that was positioned in the center of the screen. This stimulus setup was chosen as it has been shown that tasks involving multisensory integration are optimized when visual stimuli are displayed in peripheral vision (Shams et al., 2002). Auditory stimuli were in a form of a 3500 Hz pure tone. The visual and auditory stimuli had stimulus onset asynchronies (SOAs) of either 0 ms or 59 ms, 106 ms, and 153 ms (visual stimulus leading) or −59 ms, −106 ms, and −153 ms (auditory stimulus leading). SOAs were presented randomly and not equally distributed (the veridical simultaneous condition had a 6:1 ratio to any of the other six non-simultaneous conditions). In this way, there was a random and equal likelihood of simultaneous/non-simultaneous conditions, minimizing concerns about response bias. Each trial started with the following instructions: “Please judge whether the flash and the beep are presented together or separately. Press ‘1‘ for together and press ‘2‘ for separate. Once you have made the response, feedback will be presented. Use this feedback to become better at determining whether the flash and the beep occur together or separately. Press any key to begin.” Thereafter, participants were presented with the white fixation cross displayed in the center of the monitor for 500 ms. Once the fixation cross disappeared, either visual stimulus, auditory stimulus, or both stimuli together were presented. After the presentation of the stimuli, the following reminder message appeared: “Press ‘1‘ for together. Press ‘2‘ for separate.” and was displayed until the response was made. Once the response was made, participants were presented with either the phrase ‘Correct‘, in green, or ‘Incorrect‘, in red, for 1500 ms. Thereafter, the next trial began. The task consisted of 900 trials and was divided into three equal-sized blocks. A 1-min rest break was provided between the blocks.

### 2.5. Procedure

#### 2.5.1. Experimental sample

**Figure 4:**
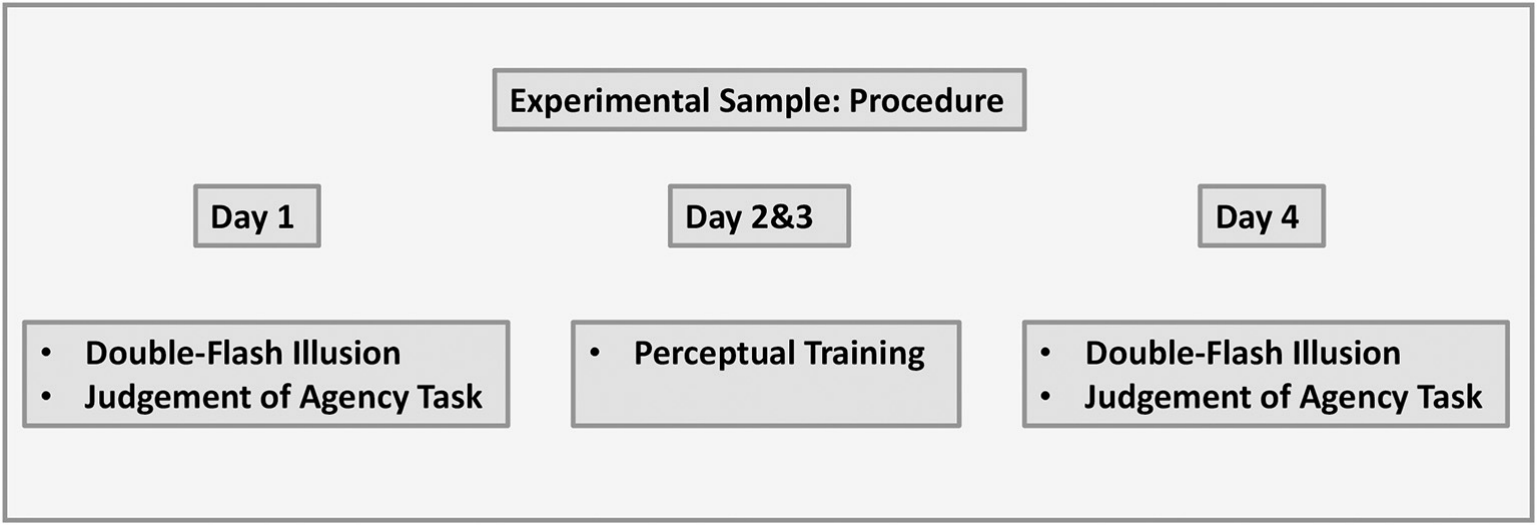
Experimental sample: procedure.

Participants completed the study online on their own IT devices by following the link provided by the researcher. The study took place over four consecutive days. Participants were instructed to ensure that they were in a dimly lit room, were reminded to adjust the volume on their devices to a comfortable hearing level, and were asked to sit ~60 cm away from the computer screen with the plane of their eyes aligned to the center of the monitor. Participants completed the double-flash illusion task and judgment of agency task on day 1 and day 4. Perceptual training was completed on day 2 and day 3. The visual representation of the data is shown in Figure 4.

#### 2.5.2. Control sample

**Figure 5:**
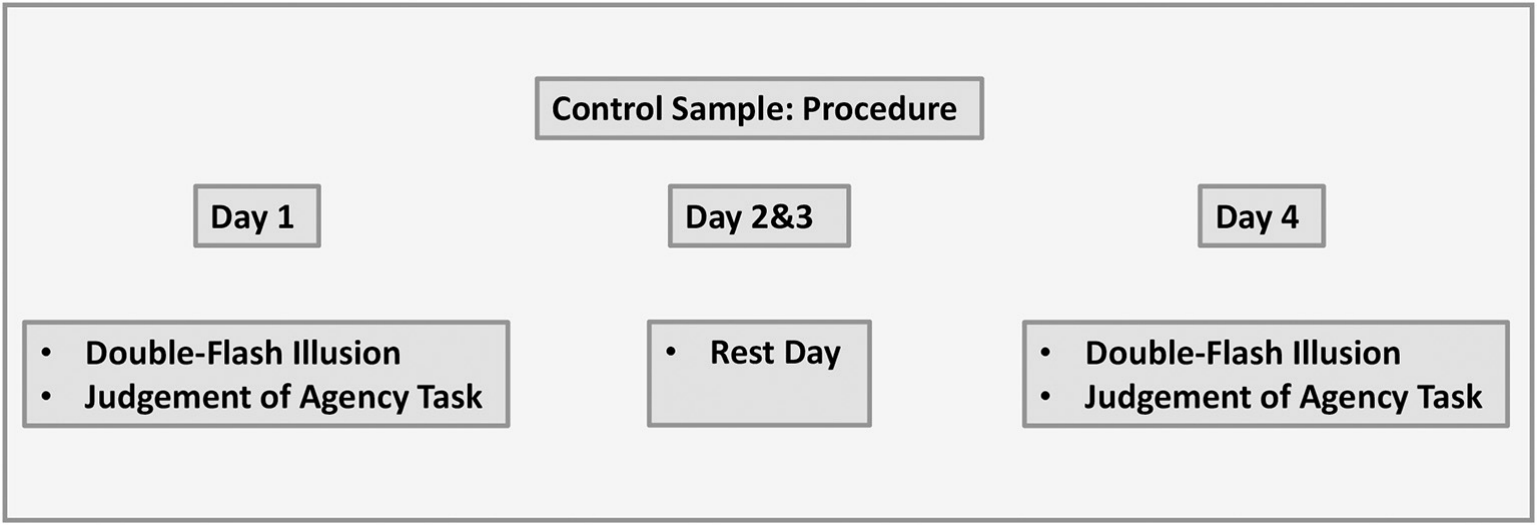
Control sample: procedure.

The protocol was the same as with the experiment sample with only one difference, that is, participants did not complete perceptual training and instead had rest days on day 2 and day 3. The visual representation of the data is shown in Figure 5.

### 2.6. Data analysis

Researchers argue that perceptual training shifts the point of subjective simultaneity (PSS) (the discrepancy at which two stimuli are perceived as simultaneous) toward the optimum (Powers et al., 2009; Stevenson et al., 2013; De Niear et al., 2018; Zerr et al., 2019). The argument is that by being provided feedback on their judgment, individuals learn what constitutes simultaneous and non-simultaneous stimuli. As such, individuals develop a better ability to discriminate between stimuli in time. This is reflected in the psychometric curve where the inflection point of the psychometric function (the point on the curve in which the concavity changes), corresponding to PSS, is taken as the width of the temporal binding window and SoA window. Additionally, the slope of the psychometric curve shows variability in the responses of PSS, with a steeper slope indicating less variability in the responses of PSS and vice versa. Therefore, the sigmoid function was used for the analysis of the SoA and TBW.

To assess the width of the TBW, the time window in which the illusion was maximally perceived, and the percentage of trials where two flashes reported were first plotted as a function of the inter-beep delay. A psychometric sigmoid function was then fitted to the data. The sigmoid function was defined by the equation:


(1) $$y=a+b/(1+exp\left(-\frac{x-c}{d}\right))),$$

Where a = upper asymptote; b = lower asymptote; c = inflection point; and d = slope. For each participant, c was taken as the TBW, i.e., the point of decay of the illusion (Cecere et al., 2015).

To examine the SoA window, the number of times the individual reported that they caused the circle to appear on the screen was recorded. Thereafter, a sigmoid function was fitted to the data, determining each participant‘s inflection point (corresponding to the width of the SoA window), in ms. A decreasing sigmoid function was used to fit the distribution of responses and was defined by Equation 1 (a = upper asymptote; b = lower asymptote; c = inflection point; d = slope). For each participant, c was taken as the SoA window, i.e., the point of decay of the self-attribution (Sato, 2009; Shimada et al., 2010).

Matlab R2020a (MathWorks) with Curve Fitting Toolbox was used to fit the psychometric functions, with statistical analysis conducted in SPSS 25 (IBM).

## 3. Results

### 3.1. Effect of perceptual training on SoA

**Figure 6:**
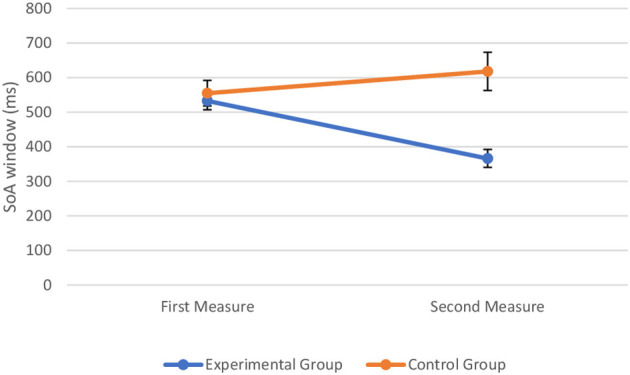
Width of the SoA window (in milliseconds) at the first and second measure when perceptual training was absent (control group) and undertaken (experimental group). The markers represent mean values along with the standard errors of the mean across participants in the experimental and control groups, respectively.

To assess the effect of perceptual training on the SoA window, we combined the data from the experimental sample and control sample to conduct a two-way mixed ANOVA with time (first measure vs. second measure) and group type (experimental vs. control) as the two factors. The assumptions of ANOVA were met. The Box's test of equality of covariance matrices showed that covariance matrices across the groups were equal, Box's M = 3.3, *p* = 0.374. The Levene's test of equality of error variances showed that the error variance was equal across groups for the SoA window at pre-perceptual training, *F*_(1, 62)_ = 1.42, *p* = 0.238 and at post-perceptual training, *F*_(1, 62)_ = 0.89, *p* = 0.351. The Shapiro–Wilk test showed that all the variables were normally distributed (*p* > 0.05). The two-way mixed ANOVA showed that there was a significant main effect of time on the width of the SoA window, *F*_(1, 62)_ = 5.26, *p* = 0.025, = 0.078. Similarly, there was a significant main effect of group type on the width of the SoA window, *F*_(1, 62)_ = 7.80, *p* = 0.007, = 0.112. There also was a significant interaction between time and group type, *F*_(1, 62)_ = 26, *p* < 0.001, = 0.295, indicating that the change in the SoA window at the first measure and second measure was significantly different in the control group and experimental group. The visual representation of the data is shown in Figure 6.

**Figure 7:**
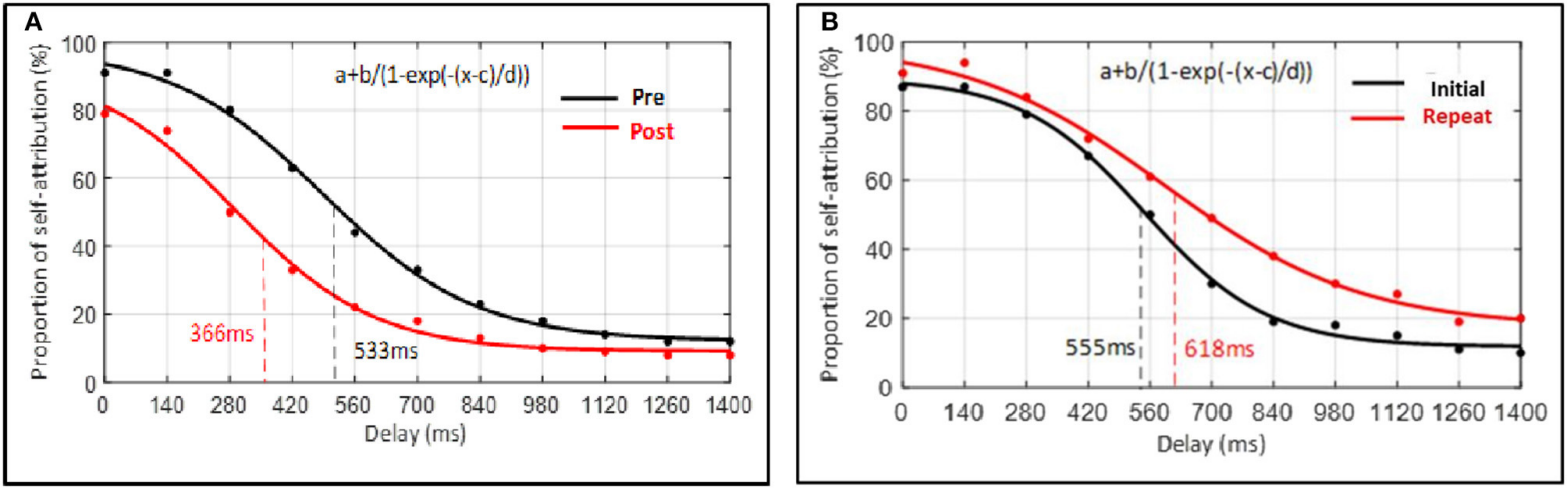
Average percentage of probability of self-attribution plotted as a function of delay. Data of each participant were averaged for each delay and, hereafter, across all participants according to the delays. Data points represent the group's average raw data. Curves represent the sigmoid fit determining the point when the SoA judgment changes, corresponding to the width of the SoA window. **(A)** Experimental condition. **(B)** Control condition.

*Post hoc* comparisons (without correction) were then conducted. A paired-sample *t*-test indicated that when perceptual training was undertaken the width of the SoA window at post-perceptual training was decreased (*M* = 366, SD = 181) compared to pre-perceptual training (*M* = 533, SD = 186), *t*_(48)_ = 7.70, *p* < 0.001, Cohen's d = 0.91. More precisely, findings showed that the inflection point, corresponding to the width of the SoA window, was lower following perceptual training (see Figure 7A). In contrast, the paired-sample *t*-test indicated that when perceptual training was absent, the width of the SoA window did not differ significantly between the initial measure (*M* = 555, SD = 143) and the repeated measure (*M* = 618, SD = 212) of the judgment of agency task, *t*_(14)_ = −1.56, *p* = 0.141, d = 0.40. Findings showed that the inflection point, corresponding to the width of the SoA window, did not differ significantly between both measures (see Figure 7B).

To further test the relative support for the null vs. alternative hypothesis, we also computed Bayes factors for the above contrasts, which revealed support for the difference in SoA window pre- and post-perceptual training (BF < 0.001, null vs. alternative), but inconclusive evidence for the comparison between the first and second time point for participants in the control condition (BF = 1.76, null vs. alternative).

### 3.2. Effect of perceptual training on TBW

**Figure 8:**
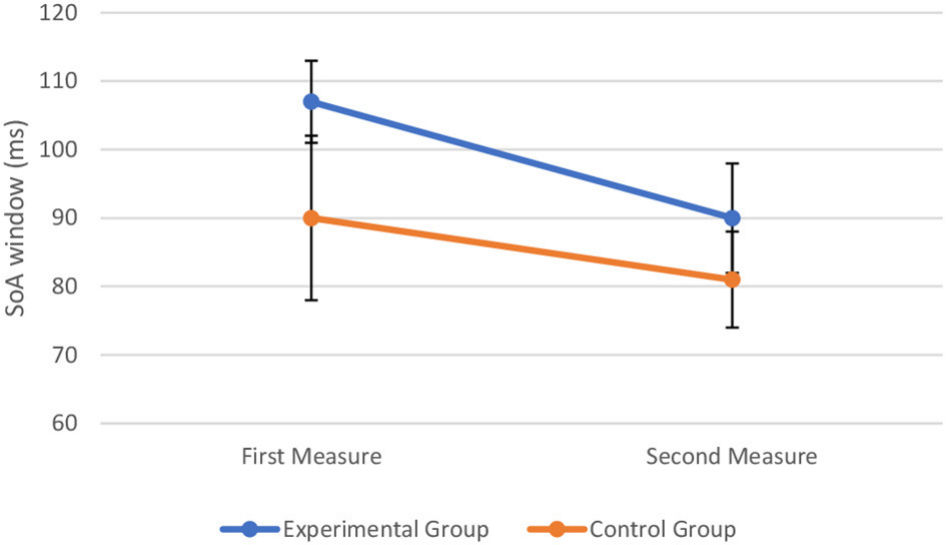
Width of the TBW (in milliseconds) at the first and second measure when perceptual training is absent (control group) and undertaken (experimental group). The markers represent mean values along with the standard errors of the mean across participants in the experimental and control groups, respectively.

To assess the effect of perceptual training on TBW, we combined the data from the experimental sample and control sample to conduct a two-way mixed ANOVA with time (first measure vs. second measure) and group type (experimental vs. control) as the two factors. The assumptions of ANOVA were met. The Box's test of equality of covariance matrices showed that covariance matrices across the groups were equal, Box's M = 6.64, *p* = 0.111. The Levene's test of equality of error variances showed that the error variance was equal across groups for TBW at pre-perceptual training, *F*_(1, 28)_ = 0.676, *p* = 0.418, and at post-perceptual training, *F*_(1, 28)_ = 1.729, *p* = 0.199. The Shapiro–Wilk test showed that all the variables were normally distributed (*p* > 0.05). The two-way mixed ANOVA showed that there was a significant main effect of time on the width of the TBW, *F*_(1, 28)_ = 4.25, *p* = 0.049, = 0.132, There is no significant main effect of group type on the width of the TBW, *F*_(1, 28)_ = 1.60, *p* = 0.217, = 0.054. There was also no significant interaction between time and group type, *F*_(1, 28)_ = 0.43, *p* = 0.519, = 0.015, indicating that the change in TBW at the first measure and second measure was not significantly different in the control group and experimental group. The visual representation of the data is shown in Figure 8.

**Figure 9:**
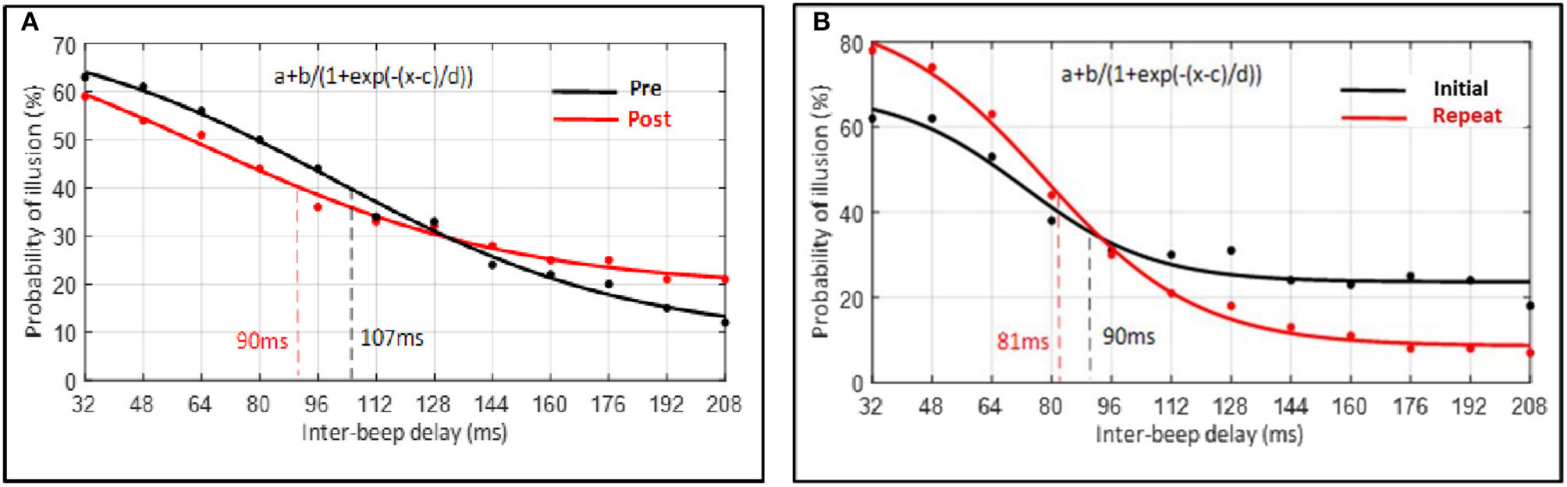
Average percentage of probability of illusion plotted as a function of inter-beep delay. Data of each participant were first averaged for each inter-beep delay and, hereafter, across all participants according to the inter-beep delays. Data points represent the group's average raw data. Curves represent the sigmoid fit determining the point of decay of the illusion, corresponding to the width of the TBW. **(A)** Experimental condition. **(B)** Control condition.

Despite the non-significant interaction, *post hoc* comparisons (without correction) were conducted to further explore the data. A paired-sample *t*-test indicated the width of the TBW to be reduced post-perceptual training (*M* = 90, SD = 36) compared to pre-perceptual training (*M* = 107, SD = 26), *t*_(19)_ = 2.23, *p* = 0.038, d = 0.55. That is, the inflection point, corresponding to the width of the TBW, was lower following perceptual training (see Figure 9A). In contrast, in the control condition, a paired-sample *t*-test indicated that the width of the TBW did not differ significantly between the initial (*M* = 90, SD = 41) and the repeated measure (*M* = 81, SD = 23) of the double-flash illusion task, *t*_(9)_ = 0.91, *p* = 0.385, d = 0.29. That is, findings showed that the inflection point, corresponding to the width of the TBW, did not differ significantly between both measures (see Figure 9B).

To further test the relative support for the null vs. alternative hypothesis, we also computed Bayes factors for the above contrasts, which revealed only weak support for the difference in TBW pre- and post-perceptual training (BF = 0.69, null vs. alternative) and weak support for the null hypothesis in the control condition (BF = 2.94, null vs. alternative).

## 4. Discussion

The main aim of the current study was to examine whether perceptual training affects TBW and SoA similarly. That is, whether the width of the TBW and the width of the SoA window reduces following perceptual training. Additionally, the current study aimed to replicate previous results concerning TBW by introducing a clearer experimental design with a control group.

First, when considering findings regarding TBW it must be stated that non-significant interaction was observed. That is, the change in TBW at the first measure and second measure was not significantly different in the control group and experimental group. Hence, making it difficult to state with confidence that perceptual training rather than completing the double-flash illusion task two times caused the reduction in the TBW. Nevertheless, it seems that such results are more likely to be due to the small sample size (A sensitivity power analysis showed that our sample size had 80% power to detect small effect sizes for two-way ANOVA (f = 0.15; α = 0.05, two-tailed). Thus, indicating underpowered study.). It appears that the reduction of TBW in the experimental group was not strong enough to lead to a significant difference between the experimental group and the control group. The abovementioned conclusion is further supported by the results showing that TBW is reduced following perceptual training with such reduction not being observed without perceptual training. However, to rule out that completing the double-flash illusion two times caused the reduction of the TBW rather than perceptual training, we urge further research to replicate this study with a larger sample.

With regard to the previous literature, the current results showing that TBW is reduced following perceptual training with such reduction not being observed without perceptual training support the predominant stance of the existing literature (Powers et al., 2009; Stevenson et al., 2013; De Niear et al., 2018; Zerr et al., 2019). More precisely, Stevenson et al. (2013) and De Niear et al. (2018) showed a reduction in TBW immediately after perceptual training, whereas, Powers et al. (2009) and Zerr et al. (2019) extended these findings to the stable reduction of TBW at least a week after the perceptual training. However, given that the above studies did not include the control condition it is unclear if the reduced TBW was observed due to perceptual training or due to the completion of the double-flash illusion task twice. The current study introduced the control condition whereby perceptual training was replaced with rest days and found that in the control condition, a reduction effect in TBW was not observed. Hence, the current findings not only support the previous literature on the topic but also strengthen the reliability of previous findings. That is, the current findings allow us to argue that perceptual training led to the reduction effect in TBW and not the repeated completion of the double-flash illusion task. As noted above, this should be replicated with a larger sample size.

The current study showed that the SoA window is reduced following perceptual training. Such a reduction was not observed in the control group where participants did not receive perceptual training. This is in line with the previous literature claiming that SoA is in part generated by temporal cues. The current study extends these findings to show a causal effect of perceptual sensitivity on the sense of agency. As previously discussed, most actions and outcomes contain some delay. Even simple action–outcome event such as pressing a switch to turn on a light involves certain delays. Despite such delays, SoA is experienced. Farrer et al. (2013) showed that if the action and its sensory outcome occur within a specific temporal interval, during which the action and the outcome are integrated, one experiences agency for the action and the outcome. However, Kawabe et al. (2013) indicated that the temporal grouping of sensory events can influence the experience of SoA. The current study extends these previous findings to show that training temporal sensitivity can affect how participants use temporal cues in attributing agency to their actions and associated outcomes.

The novel findings presented here also suggest that perceptual training not only affects lower cognitive processes (i.e., TBW) but also higher cognitive processes (i.e., SoA). This may ultimately have implications for disorders in which higher cognitive processes (that are based on temporal sensitivity) are impaired. For example, SoA has been shown to be impaired in schizophrenia spectrum disorders (Hauser et al., 2011; Krugwasser et al., 2022). That is, individuals with schizophrenia spectrum disorders often experience exaggerated self-attribution to external events. Such exaggerated self-attribution to external events leads to confusion between internally induced and externally induced sensations and can result in subjective experiences (Hauser et al., 2011; Krugwasser et al., 2022). That is, when a person has exaggerated self-attribution of external events, they may feel ownership of someone else‘s actions and their associated outcomes. For example, a person believes that an externally induced sensation (i.e., the memory of someone‘s speech) is an internally induced sensation (one‘s current thoughts). If this memory is attributed to a person‘s current thoughts, this is contradicting one‘s beliefs and expectancies. Hence, the person experiences that another (external) entity is controlling or inserting thoughts. While previous research (Norton et al., 2011) has shown that perceptual training can improve visual–motion perception in schizophrenia, future studies might extend this to the investigation of whether perceptual training might also influence delusions of agency experienced in this disorder. Disturbances in the sense of agency are also characteristic of other disorders such as anosognosia for hemiplegia and obsessive–compulsive disorder (Moore, 2016), while differences in agency processing are also observed in autistic children (Russell and Jarrold, 1999) and adults (Zalla et al., 2015). Future research should explore whether perceptual training might also alter agency processing in such populations.

As can be seen, the novel findings in relation to the SoA carry significant theoretical and practical implications. First, enhancement seen in SoA following perceptual training strongly suggests that temporal cues are more important in SoA than previously assumed. Second, these findings allow us to claim that perceptual training not only affects lower cognitive processes (i.e., TBW) but also higher cognitive processes (i.e., SoA). This, in turn, lays the path for perceptual training potentially being used to improve specific symptoms in disorders in which higher cognitive processes (that are based on temporal sensitivity) are impaired.

## 5. Limitations

Finally, it should be brought to the reader‘s attention that the current study has some methodological limitations whose discussion not only allows for the assessment of the current study but also enables to avoid similar limitations in future research. In particular, the current study excluded a large number of participants due to the experimental setup of the double-flash illusion. When tasks were completed online using Mac devices, stimuli distortion took place, such that only one tone was presented in the double-flash illusion. This meant that participants could not experience the illusion. One of the ways to resolve this technical issue would be to rewrite the Matlab script to accommodate Mac devices. Additionally, many participants were excluded due to data not fitting the sigmoid function. Other methods of analysis could be conducted in future studies that will allow to include such data. For example, recently, Buergers and Noppeney (2022) analyzed the data using sensitivity (d'), which is an index for a temporal resolution (precision) of a sensory system. Furthermore, we found that the double-flash illusion was perceived at a rate of ~20% even in the 208 ms inter-beep delay condition. This might have affected how well the data fits with the sigmoid function and as such contributed to the large data exclusion rate. We suggest that in future studies, longer inter-beep delays are used (in which participants no longer perceive the illusion). The exclusion of so many participants in the current study means that the final sample size was extremely small, and, therefore, the results should be treated with caution.

Another limitation that needs to be mentioned relates to the judgment of agency tasks. During debrief, the majority of the participants disclosed that in the 0ms delay condition they assumed that it was most likely that it was the computer that had triggered the circle to appear because it was experienced as appearing too soon to be caused by their button press. This complicates the analysis because this makes it more difficult to accurately fit a sigmoid function to the data. The reason behind the inclusion of 0 ms delay was that this condition allows to show correct identification of causing an outcome. However, the condition of 0 ms delay does not relate to temporal sensitivity. Given the complications this condition creates and not being related to temporal sensitivity, we suggest excluding such a condition from future studies.

It should also be noted that the current study used only retrospective sensitivity power analysis, which may be of limited value (Zhang et al., 2019). As such, it is difficult to make very strong claims about which effects may have been suitable or underpowered, but the final sample size in the current study (partly caused by the technical limitations described above) means that it is highly likely that the study was underpowered. This is also supported by the observation that the Bayesian analysis provided inconclusive evidence for both the null hypothesis and the alternative hypothesis. Future studies should attempt to replicate these findings with a larger sample size.

## Data availability statement

The datasets presented in this study can be found in online repositories. The names of the repository/repositories and accession number(s) can be found at: OSF: https://osf.io/u4x8v/?view_only=7802a3d16c8d4ad19044996769b704fa&lt;/b&gt.

## Ethics statement

The studies involving human participants were reviewed and approved by the University of Essex‘s Faculty Ethics Subcommittee. The patients/participants provided their written informed consent to participate in this study.

## Author contributions

AV and GH: conceptualization, methodology, software, formal analysis, investigation, and writing—original draft. PG: software. All authors contributed to the article and approved the submitted version.
